# Heme, copper, and a new way to kill cancer cells

**DOI:** 10.70401/EXO.2026.0004

**Published:** 2026-03-19

**Authors:** Xi Zhao, Li Zhuang, Boyi Gan

**Affiliations:** Department of Experimental Radiation Oncology, The University of Texas MD Anderson Cancer Center, Houston, TX 77030, USA.

**Keywords:** Heme, cuproptosis, copper, acute myeloid leukemia, BTB and CNC homology 1

## Abstract

Heme homeostasis influences mitochondrial metabolism and leukemia stem cell biology in acute myeloid leukemia. Lewis *et al.* uncover a surprising metabolic vulnerability in acute myeloid leukemia: suppression of heme biosynthesis primes leukemic cells for cuproptosis, a form of copper-dependent cell death. By linking heme depletion to mitochondrial cytochrome c oxidase (Complex IV) dysfunction, copper accumulation, and cuproptosis, the study integrates transcriptional regulation, mitochondrial metabolism, and metal homeostasis into a unified framework for selective cancer cell killing.

## 1. A New Metabolic Achilles’ Heel in Leukemia

**Figure 1.**
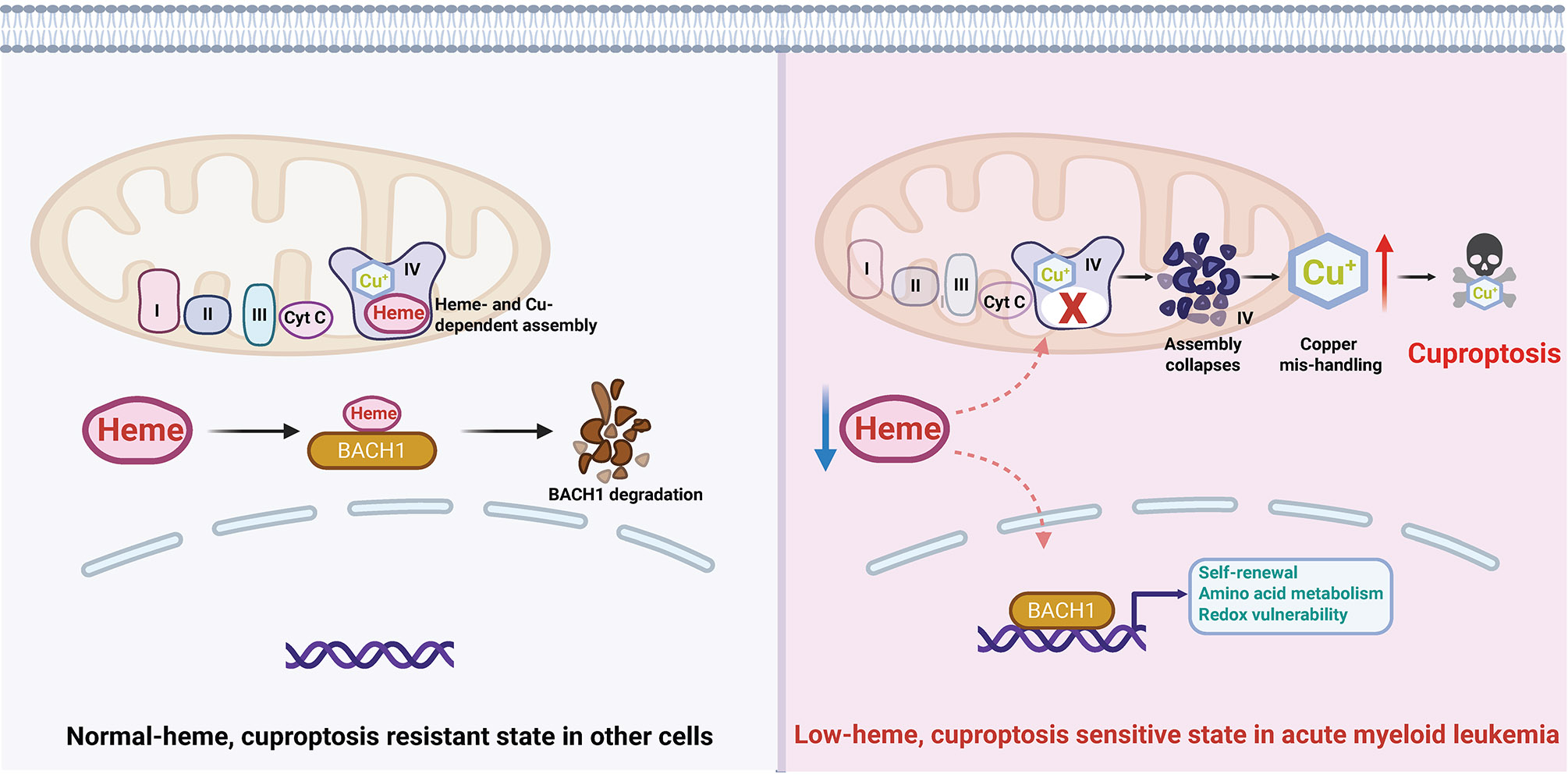
Heme depletion licenses cuproptosis in acute myeloid leukemia. Schematic model illustrating how suppression of de novo heme biosynthesis creates a metabolic vulnerability to cuproptosis in AML. On the left: normal-heme cells maintain intact Complex IV activity, balanced copper homeostasis, BACH1 degradation, and resistance to cuproptotic cell death. On the right: in contrast, in AML cells, coordinated downregulation of heme biosynthesis enzymes results in a low-heme state that stabilizes the heme-sensitive transcription factor BACH1, promoting self-renewal programs, altered amino acid metabolism, and increased redox vulnerability. Further heme depletion disrupts mitochondrial Complex IV, a respiratory complex that requires both heme and copper cofactors for proper assembly and function. Collapse of Complex IV perturbs mitochondrial copper handling, leading to intracellular copper accumulation and triggering cuproptosis. The figure was created with BioRender. AML: acute myeloid leukemia; Complex IV: cytochrome c oxidase; BACH1: BTB and CNC homology 1.

Metabolic reprogramming has long been recognized as a key feature of cancer^[1]^, yet new research continues to uncover unanticipated vulnerabilities rooted in the altered handling of essential metabolites. In a recent finding published in *Cell*^[2]^, Lewis *et al*. identify such a liability in acute myeloid leukemia (AML): a dependence on de novo heme biosynthesis that, when disrupted, triggers cuproptosis, a recently discovered copper-dependent form of regulated cell death^[3]^. By revealing how heme deprivation collapses mitochondrial cytochrome c oxidase (Complex IV), perturbs copper homeostasis, and induces lethal aggregation of lipoylated proteins, this work integrates multiple aspects of cancer metabolism into a strikingly cohesive framework (Figure 1).

Heme is best known for its role in oxygen transport in erythrocytes, but in most mammalian cells it also functions as a multifunctional cofactor and signaling molecule, regulating mitochondrial respiration, redox balance, iron metabolism, and transcriptional programs^[4]^. Unlike many metabolites, heme can be obtained either by salvage or through a multi-step biosynthetic pathway that spans mitochondria and cytosol^[4]^. Lewis *et al.* demonstrate that this biosynthetic route, rather than salvage, is a selective dependency in AML, especially within leukemic stem cells (LSCs), which exhibit coordinated downregulation of heme biosynthesis enzymes and correspondingly low intracellular heme levels^[2]^. This observation suggests that leukemia cells operate with just enough heme to survive; this low-heme state appears to support their stem-like behavior, but it also makes them especially vulnerable when heme levels drop further. Such “metabolic cliff edges” are increasingly recognized as therapeutic opportunities, where modest interference can push cancer cells beyond their capacity to survive, while sparing normal tissues.

## 2. Heme as a Transcriptional and Metabolic Rheostat

A particularly compelling aspect of the study is the demonstration that heme is not merely a metabolic substrate but also a regulator of gene expression. The authors focus on BTB and CNC homology 1 (BACH1), a heme-sensitive transcription factor^[5]^. When heme levels fall, BACH1 is stabilized and accumulates on chromatin, regulating transcriptional programs toward self-renewal, altered amino acid metabolism, and redox vulnerability^[5]^. This provides a plausible mechanistic explanation for why low-heme AML cells display LSC-like features and resistance to differentiation. Importantly, these transcriptional responses likely represent adaptive consequences of moderate heme depletion; as discussed later, cuproptosis appears to occur only when heme levels decline further and mitochondrial Complex IV collapses, leading to copper accumulation and activation of the cuproptotic death program. This observation suggests that leukemia cells may operate within a narrow “low-heme window,” in which partial heme depletion promotes BACH1-dependent stemness programs while further reductions cross a metabolic threshold that disrupts mitochondrial Complex IV and triggers cuproptosis.

In this sense, heme acts as a rheostat that integrates metabolic state with cell fate decisions. By tuning BACH1 activity, leukemia cells appear to couple mitochondrial metabolism to transcriptional identity. The finding that pharmacologic hemin can partially reverse these gene expression changes underscores the causal role of heme itself, rather than secondary metabolic effects. Importantly, these transcriptional consequences extend beyond stemness. The authors show that low-heme states sensitize cells to inhibitors of mitochondrial respiration, ferroptosis-inducing agents, and iron-sequestering drugs (with iron restriction acting here to exacerbate heme deficiency, rather than as a ferroptosis inhibitor)^[2]^. These findings therefore position heme not only as a driver of cell identity but also as a predictive biomarker for drug sensitivity, a concept that could be highly valuable in the context of AML heterogeneity.

Another intriguing implication of these findings is the potential interplay between cuproptosis and other forms of metabolically regulated cell death, particularly ferroptosis (a form of regulated cell death induced by lipid peroxidation). The BACH1-dependent transcriptional programs induced by low-heme states influence amino acid metabolism and glutathione pathways, both of which are central regulators of ferroptosis sensitivity in cancer cells^[6]^. In this context, reduced heme availability could simultaneously promote copper-dependent proteotoxic stress and weaken antioxidant defenses that normally suppress lipid peroxidation. Such conditions may create a shared biochemical environment characterized by mitochondrial dysfunction and redox imbalance. Whether these vulnerabilities operate independently, additively, or synergistically in low-heme cancers remains an open question that merits further investigation.

## 3. A Surprising Link between Heme Deprivation and Cuproptosis

The most unexpected and conceptually transformative discovery in this work is that heme deprivation induces cuproptosis. Cuproptosis, first described in 2022, is a recently defined form of regulated cell death that is triggered by intracellular copper overload and is mechanistically distinct from apoptosis, ferroptosis, and necroptosis^[3]^. Rather than relying on caspases, lipid peroxidation, or death receptor signaling, cuproptosis is initiated when copper directly binds to lipoylated mitochondrial proteins, such as the E2 subunit of the pyruvate dehydrogenase complex, dihydrolipoamide S-acetyltransferase (DLAT), causing their oligomerization, proteotoxic stress, and ultimately cell death^[3,7]^.

To uncover the mechanism by which heme deprivation kills AML cells, Lewis *et al.* performed an unbiased screen in AML cells using a metabolism-focused sgRNA library. This approach allowed them to systematically identify genes whose deletion either sensitized or protected cells from pharmacologic inhibition of heme biosynthesis. Strikingly, the strongest protective hits were genes involved in lipoic acid synthesis, mitochondrial protein lipoylation, and related factors^[2]^. Because lipoylated mitochondrial enzymes are the known substrates of copper-induced aggregation in cuproptosis^[3]^, these results immediately suggested a connection between heme starvation and this newly described cell death pathway.

Consistent with this hypothesis, the authors show that heme depletion induces multiple hallmark features of cuproptosis, including oligomerization of lipoylated DLAT, accumulation of intracellular copper, and protection from cell death upon genetic disruption of the lipoylation machinery^[2]^. Together, these findings establish that heme starvation does not merely impose generic metabolic stress, but instead funnels AML cells into a specific, copper-dependent death program. More broadly, it demonstrates how perturbations in one metabolic axis can unmask vulnerabilities in another.

## 4. Mitochondrial Complex IV as the Missing Link

How does heme depletion lead to cuproptosis? The authors trace this effect to the mitochondrial electron transport chain, specifically Complex IV. Complex IV is unique among respiratory complexes in that it requires both heme and copper cofactors for proper assembly and activity^[8]^. When heme biosynthesis is inhibited, Complex IV assembly collapses, leading to impaired respiration and destabilization of copper-handling machinery (Figure 1).

This disruption appears to cause copper ions to accumulate aberrantly within mitochondria and the cytosol, overwhelming buffering systems and triggering cuproptotic cell death^[2]^. The study thus positions Complex IV as a critical hub linking heme availability, copper homeostasis, and cell death decisions (Figure 1). This finding also reframes how we think about mitochondrial dysfunction in cancer. Rather than a generic stress signal, specific defects in cofactor assembly can channel cells into distinct death pathways. It will be interesting to explore whether other mitochondrial cofactor imbalances, such as those involving iron-sulfur clusters or flavins, might similarly control unique forms of cell death.

Although the study strongly implicates Complex IV collapse as a key proximal event linking heme depletion to copper dysregulation, additional mechanisms may also contribute. The authors provide evidence of copper overload, including increased copper in both cytosolic and mitochondrial fractions, redistribution of the copper exporter ATP7A, and reduced copper chaperone for superoxide dismutase (CCS), a marker of elevated intracellular copper, whereas the copper importer SLC31A1/CTR1 was not significantly altered. These findings suggest that defective copper sequestration, trafficking, or efflux, rather than increased uptake alone, may participate in the response to heme loss. In addition, the authors note reduced Fe–S cluster proteins under heme-depleted conditions, raising the possibility that disruption of Fe–S biology may further exacerbate mitochondrial dysfunction or proteotoxic stress. More broadly, the observed increase in HSP70 and the partial rescue by caspase inhibition suggest that mitochondrial stress responses and secondary apoptotic features may also accompany cuproptosis in this setting. Thus, while Complex IV appears to be the central mechanistic hub identified in this study, the full pathway linking heme depletion to copper overload and cell death is likely to involve a broader network of mitochondrial quality-control and metal-handling processes.

## 5. Therapeutic Implications and Selectivity

From a translational perspective, the most exciting aspect of this work is the demonstration that heme biosynthesis inhibition selectively impairs AML cells while sparing normal hematopoietic progenitors. This selectivity likely reflects the fact that normal cells maintain higher baseline heme levels and greater metabolic flexibility, allowing them to buffer transient heme loss.

*In vivo* experiments further bolster the therapeutic promise. Genetic disruption of heme biosynthesis enzymes slows leukemia progression and prolongs survival in mouse models. Importantly, the authors show that AML cells can eventually escape such pressure, underscoring the need for combination strategies^[2]^.

More broadly, the emerging concept of therapeutically exploiting cuproptosis has begun to attract significant interest as a novel anticancer strategy^[9]^. One particularly intriguing possibility is to combine heme biosynthesis inhibitors with copper ionophores, thereby amplifying cuproptotic stress. Alternatively, targeting transcriptional programs regulated by the heme–BACH1 axis could synergize with metabolic interventions. Because BACH1 controls genes involved in amino acid transport, glutathione metabolism, and redox regulation, perturbing these transcriptional networks may weaken cellular buffering capacity and thereby lower the threshold for copper-induced mitochondrial stress and cuproptosis. The study also hints at potential synergy with ferroptosis inducers and mitochondrial inhibitors, pointing toward a broader network of vulnerabilities centered on redox and mitochondrial homeostasis. More broadly, it will be interesting to explore whether targeting heme metabolism could synergize with existing treatment modalities, such as chemotherapy or immunotherapy, particularly given emerging evidence that metabolic cell death pathways can shape anti-tumor immune responses^[7]^.

While these findings highlight an attractive therapeutic vulnerability in AML, the systemic inhibition of heme biosynthesis raises important considerations. Heme is essential for numerous physiological processes, particularly in mitochondria-rich tissues such as the liver, heart, and skeletal muscle, where it supports respiratory chain function and other heme-dependent enzymes. Consequently, sustained systemic suppression of heme synthesis could potentially compromise mitochondrial function in normal tissues. At present, the feasibility and safety of pharmacologically targeting heme biosynthesis in patients remain uncertain. Nevertheless, the preclinical data presented by this study provide encouraging indications of a potential therapeutic window. In particular, normal hematopoietic progenitors were substantially more resistant than AML cells to pharmacologic inhibition of heme biosynthesis, suggesting that leukemic cells may operate closer to a critical threshold of heme availability. These observations raise the possibility that transient or partial inhibition of heme biosynthesis, or combination strategies that amplify cuproptotic stress, could selectively target leukemia cells while minimizing toxicity to normal tissues.

## 6. Concluding Remarks and Open Questions

Together, Lewis *et al.* provide a powerful example of how revisiting fundamental metabolic pathways can yield unexpected therapeutic insights. By uncovering a heme-dependent gateway to cuproptosis, they connect mitochondrial biology, metal homeostasis, transcriptional regulation, and cell death into a single conceptual framework. This study not only expands the biological repertoire of cuproptosis but also highlights heme metabolism as a promising and previously underappreciated target in leukemia.

This work also opens several important questions for further investigation. First, how universal is this heme–cuproptosis axis across cancers? While AML appears especially sensitive, other malignancies with low heme states or suppressed heme biosynthesis may exhibit similar vulnerabilities. Consistent with this possibility, transcriptional signatures reflecting heme biosynthesis activity may also serve as predictive biomarkers for cuproptosis sensitivity. In the current study, AML cells with lower expression of heme biosynthesis enzymes showed increased dependence on this pathway and heightened vulnerability to its inhibition, raising the possibility that such signatures could help identify certain solid tumors responsive to cuproptosis-based therapies. Second, what determines whether copper accumulation leads to cuproptosis versus other forms of stress? The requirement for lipoylated mitochondrial proteins suggests a narrow mechanistic window, but how this intersects with other mitochondrial quality control pathways remains unclear. Third, the role of heme as a signaling molecule deserves deeper exploration. Beyond BACH1, heme directly influences the activity of a range of transcription factors and signaling enzymes across biological systems, and functions as a regulatory ligand that modulates DNA binding, protein activity, and gene expression in response to cellular heme status^[10]^. Mapping these networks could reveal additional links between metabolism and cell identity. Finally, the clinical feasibility of targeting heme biosynthesis remains to be established. While tools used in this study, like succinylacetone and N-methylprotoporphyrin IX, are valuable experimental probes, the development of selective, bioavailable inhibitors will be essential for translational progress.
